# Association between statin medication and hearing impairment in a national health screening cohort

**DOI:** 10.1038/s41598-021-93916-z

**Published:** 2021-07-13

**Authors:** So Young Kim, Chang Ho Lee, Chanyang Min, Dae Myoung Yoo, Hyo Geun Choi

**Affiliations:** 1 Department of Otorhinolaryngology-Head and Neck Surgery, CHA Bundang Medical Center, CHA University, Seongnam, Korea; 2 Hallym Data Science Laboratory, Hallym University College of Medicine, Anyang, Korea; 3 Graduate School of Public Health, Seoul National University, Seoul, Korea; 4 Department of Otorhinolaryngology-Head and Neck Surgery, Hallym University College of Medicine, Anyang, Korea

**Keywords:** Diseases, Neurology, Risk factors, Signs and symptoms

## Abstract

This study aimed to investigate the association of previous stain use with hearing impairment in an adult population. Data from the ≥ 40-year-old population in the Korean National Health Insurance Service Health Screening Cohort were used. The hearing impairment group was classified based on the national registry of hearing-impaired persons. Control participants were randomly selected and matched for age, sex, income, and region of residence. The number of days of statin prescription during the 2 years before the diagnosis of hearing impairment was compared between the hearing impairment group and the control group using conditional logistic regression analysis. Additional analyses were conducted according to age and sex. The number of days of previous statin use was not different between the hearing impairment group and the control group (adjusted odds ratio [aOR] = 0.94, 95% confidence interval (CI) = 0.86–1.02, *P* = 0.118). According to age, in the ≥ 70-year-old group, those with hearing impairment had 11% lower rates of previous statin use than those in the control group (aOR = 0.89, 95% CI = 0.80–0.99, *P* = 0.039). According to sex, in the male group, 12% lower rates of previous statin use were observed among those with hearing impairment than among those in the control group (aOR = 0.88, 95% CI = 0.79–0.99, *P* = 0.037). Previous statin use might have an effect on reducing the prevalence of hearing impairment in elderly individuals and men.

## Introduction

Statins, 2-hydroy-3-methyl-glutaryl-coenzyme A reductase (HMG-CoA) inhibitors, are commonly prescribed lipid-lowering agents worldwide^1^. By competitively inhibiting HMG-CoA reductase, statins suppress a critical step of cholesterol synthesis^1^. The reduction in the activity of cholesterol synthesis pathways has therapeutic and protective effects on dyslipidemia and other cardiovascular diseases^2^. Beyond the lipid-lowering properties via HMG-CoA inhibition, statins have ancillary activities that make them beneficial to cardiovascular systems^3^. Statins directly activate the expression of vasodilators and reduce the expression of vasoconstrictors, thereby improving endothelial function^3^. In addition, inflammatory responses could be reduced by reducing the synthesis of proinflammatory molecules^3^. These pleiotropic effects of statins have broadened the possible clinical indications of statins from cardiovascular disorders to neurologic disorders such as dementia^4^ and Parkinson’s disease^5^.

Hearing impairment is a prevalent disorder whose incidence tends to increase with age and is the disease responsible for the third largest health burden worldwide (36.3 million [95% confidence interval (CI) = 25.3–50.9] years lived with disability)^6^. However, no effective therapies are available in the clinic for sensorineural hearing impairment. Both cardiovascular compromise and neural degeneration are risk factors for hearing impairment^7–9^. Because statins are suggested to be effective for cardiovascular and neural disorders, hearing impairment could be another ancillary target disorder for statins. A recent clinical study demonstrated the otoprotective effects of atorvastatin in patients treated with cisplatin for head and neck cancer^10^. A few previous studies investigated the association of statin use with sudden sensorineural hearing loss (SSNHL) and yielded controversial results^11,12^. A case–control study proposed an association of increased SSNHL risk with statin use^11^. On the other hand, our previous study demonstrated no significant association between SSNHL and statin use by attenuating possible confounding effects using a matched control group and adjusting for many cardiovascular comorbidities^12^. Moreover, there has been little clinical research on the effects of statins on hearing impairments in the general population.

We postulated that previous statin use may have a protective effect against hearing impairment. Hearing impairment in the adult population could have multiple etiologies, such as age-related degenerative processes, noise exposure, ototoxic drug exposure, smoking, adiposity, and chronic diseases^13–15^. To encompass all these types of hearing impairment, this study collected data from a nationally registered hearing-impaired population and compared the previous statin use in this population to that in the control population. Few previous studies have evaluated the impact of statin use on hearing impairment in such a large population.

## Materials and methods

### Ethics

The ethics committee of Hallym University (2019–10-023) approved this study. The requirement for written informed consent was waived by the Institutional Review Board. All analyses adhered to the guidelines and regulations of the ethics committee of Hallym University.

### Study population and participant selection

**Figure 1:**
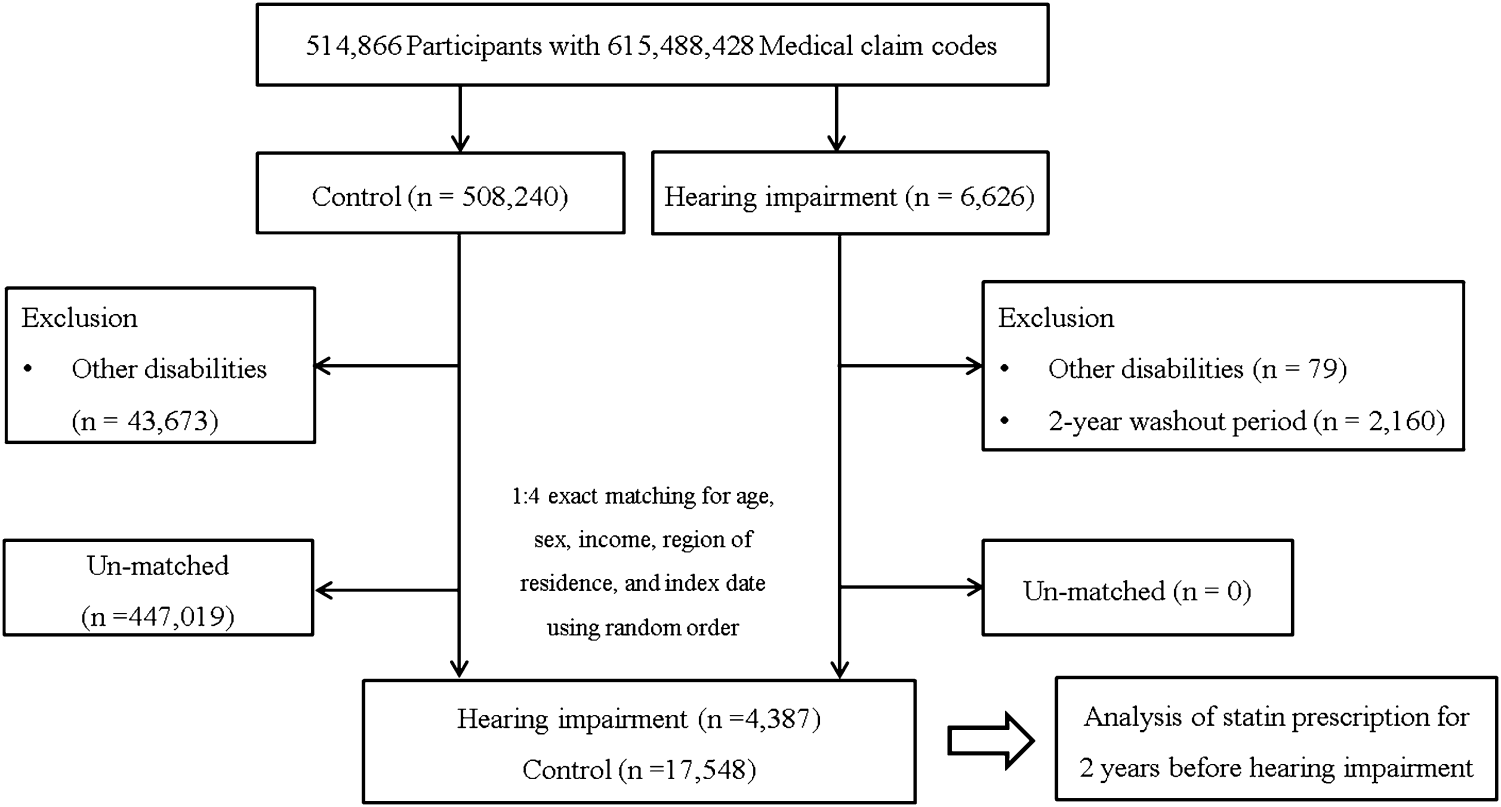
A schematic illustration of the participant selection process that was used in the present study. Of a total of 514,866 participants, 4,387 participants with hearing impairment were matched with 17,548 control participants for age, sex, income, and region of residence.

A detailed description of the Korean National Health Insurance Service-Health Screening Cohort data is described elsewhere^16^. Participants with hearing impairment were selected from 514,866 participants with 615,488,428 medical claim codes from 2002 through 2015 (n = 6,626). Individuals were included in the control group if they were not defined as having hearing impairment from 2002 through 2015 (n = 508,240). Participants who were diagnosed with other disabilities were excluded (n = 79 in the hearing impairment group, n = 43,673 in the control group). To assess the history of only the previous 2 years, we excluded participants with hearing impairment who were diagnosed with hearing impairment before 2003 (n = 2,160). Hearing impairment participants were 1:4 matched with control participants for age, sex, income, and region of residence. To minimize selection bias, the control participants were selected with a random number order. The index date of each hearing impairment participant was set as the time of diagnosis of hearing impairment. The index date of control participants was set as the index date of their matched hearing impairment participant. Therefore, each matched hearing impairment participant and control participant had the same index date. During the matching procedure, 447,019 control participants were excluded. Ultimately, 4,387 hearing impairment participants were 1:4 matched with 17,548 control participants (Fig. 1).

### Independent variable (number of days of statin medication prescription)

The number of days of statin prescription was assessed as a continuous variable for 2 years (730 days) before the index dates in both hearing impairment and control participants. The statins investigated in this study included atorvastatin, fluvastatin, lovastatin, pitavastatin, pravastatin, rosuvastatin, and simvastatin. Pravastatin and rosuvastatin were categorized as hydrophilic statins, and atorvastatin, fluvastatin, lovastatin, pitavastatin, and simvastatin were categorized as lipophilic statins.

### Dependent variable (hearing impairment)

Hearing impairment participants were defined as those who were registered as hearing impairment persons in the Ministry of Health and Welfare. Among them, we excluded participants with other disabilities. These individuals were divided into 2 groups based on the degree of impairment: severe hearing impairment (hearing threshold ≥ 60 dB in both ears; ≥ 80 dB in one ear and ≥ 40 dB in one ear) and profound hearing impairment (hearing threshold ≥ 90 dB in both ears)^17^. In Korea, to be registered as a hearing-impaired person, individuals must be checked 3 times using a pure tone audiometry test (PTA) and 1 time using an auditory brainstem response^17^. The average hearing threshold of PTA was calculated as follows: (500 Hz + 2 * 1000 Hz + 2 * 2000 Hz + 4000 Hz)/6^17^.

### Covariates

Age was divided into 5-year intervals: 40–44, 45–49, 50–54…, and 85 + years old (total of 10 age groups). Income was classified into 5 classes (class 1 [lowest income]-5 [highest income]). The region of residence was grouped into urban and rural areas following the method used in our previous study^18^. Tobacco smoking, alcohol consumption, and obesity according to body mass index (BMI, kg/m^2^) were categorized in the same way as in our previous study^19^. The records of total cholesterol (mg/dL), systolic blood pressure (SBP, mmHg), diastolic blood pressure (DBP, mmHg), fasting blood glucose (mg/dL), and hemoglobin (g/dl) were used. Missing values for fasting blood glucose (n = 2 [0.009%]), total cholesterol (n = 3 [0.013%]), and hemoglobin (n = 3 [0.013%]) were replaced by the mean values of each variable from the final group of selected participants.

The Charlson Comorbidity Index (CCI) has been used widely to measure disease burden using 17 comorbidities as a continuous variable (0 [no comorbidities] through 29 [multiple comorbidities])^20^. In our study, we excluded dementia from the CCI score.

Regarding statin medication, a dyslipidemia diagnosis (ICD-10 codes: E78) was additionally assigned if participants were treated ≥ 2 times.

### Statistical analyses

The general characteristics of the hearing impairment and control groups were compared using the chi-square test for categorical variables and the independent *t* test for continuous variables.

To analyze the odds ratios (ORs) with 95% confidence intervals (CIs) of statin prescription during a period of 1 year for hearing impairment, conditional logistic regression was used in the groups matched for age, sex, income, and region of residence. In these analyses, a crude model (simple), model 1 (SBP, DBP, fasting blood glucose, total cholesterol, hemoglobin, and dyslipidemia) and model 2 (model 1 plus obesity, smoking, alcohol consumption, and CCI scores) were used. The analysis was stratified by age, sex, income, and region of residence.

For the subgroup analyses, we divided the participants by age and sex (< 70 years old and ≥ 70 years old, men and women) to confirm these associations according to age and sex. The division of the age groups was determined by the median value of the total number of participants. Additionally, we performed analyses according to the type of statin (hydrophilic or lipophilic). We further analyzed the ORs of statin prescription over a period of 1 year for hearing impairment by severity of hearing impairment.

Two-tailed analyses were performed, and significance was defined as P values less than 0.05. SAS version 9.4 (SAS Institute Inc., Cary, NC, USA) was used for statistical analyses.

## Results

The durations of statin use were not significantly different between the hearing impairment and control groups (51.86 ± 157.1 vs. 53.59 ± 162.2 days, *P* = 0.525, Table 1). The hearing impairment group showed lower frequencies of alcohol consumption and lower levels of total cholesterol than the control group (*P* = 0.031 and *P* = 0.010, respectively). The distributions of obesity, smoking status, SBP, DBP, fasting blood glucose, hemoglobin, CCI score, and dyslipidemia were not different between the hearing impairment and control groups.

**Table 1:** General characteristics of participants.

Characteristics	Total participants		
	Hearing impairment	Control	*P *value
Total number (n, %)	4,387 (100.0)	17,548 (100.0)	
Age (years old) (n, %)			1.000
40–44	42 (1.0)	168 (1.0)	
45–49	144 (3.3)	576 (3.3)	
50–54	302 (6.9)	1,208 (6.9)	
55–59	485 (11.1)	1,940 (11.1)	
60–64	621 (14.2)	2,484 (14.2)	
65–69	788 (18.0)	3,152 (18.0)	
70–74	851 (19.4)	3,404 (19.4)	
75–79	715 (16.3)	2,860 (16.3)	
80–84	356 (8.1)	1,424 (8.1)	
85 +	83 (1.9)	332 (1.9)	
Sex (n, %)			1.000
Male	2,651 (60.4)	10,604 (60.4)	
Female	1,736 (39.6)	6,944 (39.6)	
Income (n, %)			1.000
1 (lowest)	829 (18.9)	3,316 (18.9)	
2	612 (14.0)	2,448 (14.0)	
3	693 (15.8)	2,772 (15.8)	
4	809 (18.4)	3,236 (18.4)	
5 (highest)	1,444 (32.9)	5,776 (32.9)	
Region of residence (n, %)			1.000
Urban	1,807 (41.2)	7,228 (41.2)	
Rural	2,580 (58.8)	13,320 (58.8)	
Obesity (n, %) ‡			0.617
Underweight	161 (3.7)	678 (3.9)	
Normal	1,640 (37.4)	6,470 (36.9)	
Overweight	1,131 (25.8)	4,673 (26.6)	
Obese I	1,354 (30.9)	5,290 (30.3)	
Obese II	101 (2.3)	437 (2.5)	
Smoking status (n, %)			0.066
Nonsmoker	3,182 (72.5)	12,427 (70.9)	
Past smoker	477 (10.9)	1,976 (11.3)	
Current smoker	728 (16.6)	3,145 (17.9)	
Alcohol consumption (n, %)			0.031*
< 1 time a week	3,234 (73.7)	12,651 (72.1)	
≥ 1 time a week	1,153 (26.3)	4,897 (27.9)	
Systolic blood pressure (n, %)			0.544
< 120 mmHg	1,030 (23.5)	4,130 (23.5)	
120–139 mmHg	2,076 (47.3)	8,159 (46.5)	
≥ 140 mmHg	1,281 (29.2)	5,259 (30.0)	
Diastolic blood pressure (n, %)			0.144
< 80 mmHg	1,842 (42.0)	7,166 (40.8)	
80–89 mmHg	1,634 (37.3)	6,510 (37.1)	
≥ 90 mmHg	911 (20.8)	3,872 (22.1)	
Fasting blood glucose (n, %)			0.069
< 100 mg/dL	2,692 (61.4)	10,556 (60.2)	
100–125 mg/dL	1,259 (28.7)	5,041 (28.7)	
≥ 126 mg/dL	436 (9.9)	1,951 (11.1)	
Total cholesterol (n, %)			0.010*
< 200 mg/dL	2,475 (56.4)	9,528 (54.3)	
200–239 mg/dL	1,384 (31.6)	5,647 (32.3)	
≥ 240 mg/dL	528 (12.0)	2,373 (13.5)	
Hemoglobin (mean, SD)	13.76 (1.46)	13.78 (1.47)	0.549
CCI score (n, %)			
0	2,385 (54.4)	10,186 (58.1)	
1	854 (19.5)	3,012 (17.2)	
2	536 (12.2)	1,755 (10.0)	
3	298 (6.8)	1,092 (6.2)	
≥ 4	314 (7.2)	1,503 (8.6)	
Dyslipidemia (n, %)			0.067
Yes	2,481 (56.6)	10,192 (58.1)	
No	1,906 (43.5)	7,356 (41.9)	
The dates of statin prescription			
(days, mean, SD)	51.86 (157.1)	53.59 (162.2)	0.525

Abbreviations: CCI, Charlson comorbidity index; SD, standard deviation.

*Chi-square test. Significance at P < 0.05.

^†^Independent *t* test. Significance at P < 0.05.

^‡^Obesity (BMI, body mass index, kg/m^2^) was categorized as < 18.5 (underweight), ≥ 18.5 to < 23 (normal), ≥ 23 to < 25 (overweight), ≥ 25 to < 30 (obese I), and ≥ 30 (obese II).

^§^CCI scores were calculated without dementia.

The number of days of statin use per year was not associated with hearing impairment (adjusted OR [aOR] = 0.94, 95% CI = 0.86–1.02, *P* = 0.118 in model 2, Table 2).

**Table 2:** Crude and adjusted odd ratios (95% confidence interval) of date of statin prescription (1 year) for hearing impairment with stratified subgroup according age and sex.

Characteristics	Odds ratios for hearing impairment					
	Crude†	*P *value	Model 1†‡	*P *value	Model 2†§	*P *value
**Total participants (n = 21,935)**						
Statin prescription (1 year)	0.98 (0.90–1.05)	0.521	0.94 (0.86–1.02)	0.115	0.94 (0.86–1.02)	0.118
**Age < 70 years old (n = 11,910)**						
Statin prescription (1 year)	1.04 (0.93–1.17)	0.498	1.00 (0.89–1.13)	0.959	1.03 (0.89–1.13)	0.958
**Age ≥ 70 years old (n = 10,025)**						
Statin prescription (1 year)	0.93 (0.84–1.03)	0.162	0.89 (0.80–1.00)	0.040*	0.89 (0.80–0.99)	0.039*
**Men (n = 13,255)**						
Statin prescription (1 year)	0.93 (0.84–1.04)	0.190	0.89 (0.79–0.99)	0.039*	0.88 (0.79–0.99)	0.037*
**Women (n = 8,680)**						
Statin prescription (1 year)	1.03 (0.92–1.15)	0.613	0.99 (0.88–1.12)	0.901	0.99 (0.88–1.12)	0.917

Abbreviations: CCI, Charlson Comorbidity Index;

*Conditional logistic regression analysis, Significance at P < 0.05.

^†^Stratified model for age, sex, income, and region of residence.

^‡^Model 1 was adjusted for SBP, DBP, fasting blood glucose, total cholesterol, hemoglobin, and dyslipidemia.

^§^Model 2 was adjusted for model 1 plus obesity, smoking, alcohol consumption, and CCI scores.

According to age, the older subgroup (≥ 70 years old) demonstrated a lower rate of hearing impairment associated with long durations of statin use (aOR = 0.89, 95% CI = 0.80–0.99, *P* = 0.039). According to sex, men showed a lower rate of hearing impairment related to long durations of statin use (aOR = 0.88, 95% CI = 0.79–0.99, *P* = 0.037).

According to the degree of hearing impairment, neither the severe hearing impairment nor the profound hearing impairment group showed an association of hearing impairment with the number of days of statin use (Table 3).

**Table 3:** Crude and adjusted odd ratios (95% confidence interval) of date of statin prescription (1 year) for hearing impairment by severity of hearing impairment.

Characteristics	Odds ratios for hearing impairment					
	Crude†	*P *value	Model 1†‡	*P *value	Model 2†§	*P *value
**Severe hearing loss (n = 4,075 for hearing impairment, n = 16,300 for control)**						
Statin prescription (1 year)	0.97 (0.90–1.06)	0.536	0.93 (0.86–1.01)	0.098	0.93 (0.86–1.01)	0.099
**Profound hearing loss (n = 312 for hearing impairment, n = 1,248 for control)**						
Statin prescription (1 year)	0.97 (0.68–1.41)	0.876	1.03 (0.70–1.52)	0.880	1.03 (0.69–1.53)	0.881

Abbreviations: CCI, Charlson Comorbidity Index;

*Conditional logistic regression analysis, Significance at P < 0.05.

^†^Stratified model for age, sex, income, and region of residence.

^‡^Model 1 was adjusted for systolic blood pressure, diastolic blood pressure, fasting blood glucose, total cholesterol, hemoglobin, and dyslipidemia.

^§^Model 2 was adjusted for model 1 plus obesity, smoking, alcohol consumption, and CCI scores.

Statins were divided according to hydrophilic and lipophilic types. Hydrophilic statin use was not associated with the rate of hearing impairment in the total population (S1 Table). However, in the ≥ 70-year-old group, a lower rate of hearing impairment was associated with a long duration of hydrophilic statin use (aOR = 0.77, 95% CI = 0.60 – 1.00, *P* = 0.046). Lipophilic statins were not related to the rate of hearing impairment in the total population or in any age or sex subgroups (S2 Table). According to the degree of hearing impairment, neither hydrophilic nor lipophilic statins were associated with severe or profound hearing impairment (S3 Table and S4 Table).

## Discussion

The number of days of previous statin use was not linked with hearing impairment in the adult Korean population. This study considered comorbidities, lifestyle factors, and laboratory measurements to attenuate the potential confounding effects. Although there was no association between hearing impairment and previous statin use in the overall population, elderly and male subgroups showed a negative association of previous statin use with hearing impairment. This study added to previous findings by evaluating the impacts of previous statin use on hearing impairment in a large population with objectively measured hearing impairment. Few studies have investigated the association of statin use in hearing-impaired patients with objectively measured hearing results.

Several prior studies have suggested protective effects of statin use on hearing impairment^21–23^. A double-blind, prospective, randomized clinical trial demonstrated a tendency of a protective effect of 7 months of atorvastatin medication use for tinnitus in 60- to 75-year-old patients with presbycusis (7.2-point reduction in tinnitus score in the presbycusis group vs. 2-point increase in tinnitus score in the placebo group, *P* = 0.08)^21^. Another prospective study reported a decrease in hearing thresholds at 6 kHz and tinnitus after 6 months of statin use (atorvastatin, rosuvastatin, and simvastatin) in 84 adults with hyperlipidemia^22^. The Blue Mountains Hearing Study including individuals aged ≥ 50 years old described that participants with self-reported statin use showed 48% decreased odds of hearing impairment compared with the odds in statin non-users (OR = 0.52 [95% CI = 0.29–0.93])^23^. On the other hand, a case report suggested hazardous effects of statin use on hearing impairment, although causality has not been established^24^. Bilateral, progressive, middle-frequency hearing impairment (“cookie bite”) was induced following 18 months of atorvastatin use in a 32-year-old man and three additional anonymous cases^24^. The authors of the study suggested potential side effects of coadministered drugs without sufficient supportive evidence^24^. The potential drug-drug interactions of statins with other medications and related adverse drug reactions could be linked with hearing impairment associated with atorvastatin use^25^.

A number of experimental studies have suggested otoprotective effects of statins^26–28^. The lipid-lowering effects and reduction in atherosclerosis could mediate the otoprotective effects of statins^28^. Together with the reduction in atherosclerotic lesions, hearing impairment was prevented in apolipoprotein E gene knockout mice when they were treated with simvastatin^28^. The anti-inflammatory and antioxidative responses associated with statin use could be linked with the otoprotective effects of statins^27^. Cisplatin-induced hearing loss, which is accompanied by inflammation and oxidative stress, was reduced by lovastatin administration in adult rats^27^. The investigators demonstrated that lovastatin administration induced heat shock protein expression in the cochlea, protecting hair cells against cisplatin-induced injuries^27^. Noise-induced hearing loss, which is also related to inner ear inflammation and oxidative injuries, was attenuated by a low dose of atorvastatin (5 mg/kg) in mice^26^. However, the otoprotective effects of statins could be variable according to the dose of statins. A higher dose of atorvastatin (25, 50 mg/kg) did not show otoprotective effects in noise-induced hearing loss^26^. These variations might explain the lack of a significant association of previous statin use with hearing impairment in this study.

Only the old-age group and male group showed protective effects of previous statin use in this study. The high proportions of cardiovascular comorbidities in the elderly population could mediate the risk of hearing impairments. The lipid-lowering and anti-inflammatory effects of statins might be more effective in older patients with more cardiovascular comorbidities than younger patients^29,30^. For instance, statins reduced the risk of cardiovascular events and mortality in elderly individuals with type 2 diabetes (hazard ratio = 0.76, 95% CI = 0.65—0.89)^29^. The subgroup analyses also described the protective effects of previous statin use in the male group in the present study. This negative association between previous statin use and hearing impairment in men could be attributed to the higher rate of cardiovascular risk factors, including cardiovascular diseases, obesity, and smoking, in men than in women. In addition, sex differences in adherence to statin medication might affect the association of previous statin use with hearing impairments^31^. It was reported that women showed lower compliance with statin medication than men^31^.

According to the types of statins, only hydrophilic statins showed an association with a lower rate of hearing impairment in the ≥ 70-year-old population. Hydrophilic statins have lower tissue absorption and lower dependency on cytochrome P450 metabolism than lipophilic statins, so hydrophilic statins might result in fewer adverse reactions than lipophilic statins^32^. In addition, a previous study reported superior anti-inflammatory effects of hydrophilic statins compared with lipophilic statins, with higher levels of adiponectin and lower levels of C-reactive protein in atherosclerosis patients^33^. The lower rate of side effects and greater anti-inflammatory effects of hydrophilic statins could explain the lower rate of hearing impairment in elderly individuals in the present study.

This study included a large number of participants. The initial study population was 514,844 which represented the Korean population. To minimize the potential confounder effects, the participants with missing data were excluded and matched hearing impairment and control groups were selected. Finally, the 5,487 of hearing impairment group and 17,548 of control group were analyzed. The degree of hearing impairment was based on PTA and auditory brainstem response threshold tests. Because the hearing-impaired person registry is managed by the Korean government, which provides medical cost assistance for hearing aids, the likelihood of possible misclassification was not high in the present study. In addition, the potential confounding effects were attenuated by adjusting for past medical history, smoking, alcohol consumption, obesity, blood pressure, total cholesterol, and hemoglobin. However, a few limitations should be considered when interpreting the present results. The types of hearing impairment could not be differentiated in the present study. The pathophysiology of hearing impairment could be different according to the type of hearing impairment, such as noise-induced impairment, ototoxicity, and presbycusis. Previous statin use was assessed based on prescription data in this study. Thus, compliance with the statin prescription could influence the associations in the present study. Moreover, the dose of statins could not be determined in this study. More than seven types of statins are available in the clinic and have some variations in pharmacodynamics in terms of bioavailability, cytochrome P-450-linked metabolism, and cellular transportation^1^. Last, although we considered numerous possible confounders, there might be confounding variables, such as noise exposure and stress levels, that were not assessed.

In conclusion, previous statin use was not related to hearing impairment in the adult Korean population. However, the old-age and male groups showed a relationship between previous statin use and a low rate of hearing impairment.

## Supplementary Information

Supplementary Information.
