# Supplementary material for: Association between statin medication and hearing impairment in a national health screening cohort
Source: Sci Rep. 2021 Jul 13;11:14388. doi: 10.1038/s41598-021-93916-z (PMC8277800; doi:10.1038/s41598-021-93916-z)
Supplement: Supplementary file 1 — Supplementary Information. [file 41598_2021_93916_MOESM1_ESM.pdf]

Association between statin medication and hearing impairment in a national health screening cohort

So Young Kim, MD, PhD[1], Chang Ho Lee, MD, PhD[1], Chanyang Min, PhD[2,3], Dae Myoung Yoo, MS[2], Hyo Geun Choi, MD, PhD[2,4]*

[1]Department of Otorhinolaryngology-Head & Neck Surgery, CHA Bundang Medical Center, CHA University, Seongnam, Korea

[2]Hallym Data Science Laboratory, Hallym University College of Medicine, Anyang, Korea

[3]Graduate School of Public Health, Seoul National University, Seoul, Korea

[4]Department of Otorhinolaryngology-Head & Neck Surgery, Hallym University College of Medicine, Anyang, Korea

Running title: Hearing loss and statin

***Correspondence**: pupen@naver.com

S1 table Crude and adjusted odd ratios (95% confidence interval) of date of hydrophilic statin prescription (1 year) for hearing impairment with stratified subgroup according age and sex

Characteristics	Odds ratios for hearing impairment					
	Crude†	P-value	Model 1†‡	P-value	Model 2†§	P-value
Total participants (n = 21,935)						
Hydrophilic statin prescription (1 year)	0.93 (0.77-1.12)	0.399	0.89 (0.74-1.07)	0.217	0.89 (0.74-1.07)	0.218
Age < 70 years old (n = 11,910)						
Hydrophilic statin prescription (1 year)	1.10 (0.85-1.42)	0.491	1.06 (0.82-1.38)	0.652	1.06 (0.82-1.38)	0.653
Age ≥ 70 years old (n = 10,025)						
Hydrophilic statin prescription (1 year)	0.81 (0.63-1.04)	0.098	0.78 (0.60-1.00)	0.051	0.77 (0.60-1.00)	0.046*
Men (n = 13,255)						
Hydrophilic statin prescription (1 year)	0.85 (0.66-1.09)	0.192	0.82 (0.63-1.05)	0.110	0.81 (0.63-1.04)	0.100
Women (n = 8,680)						
Hydrophilic statin prescription (1 year)	1.03 (0.79-1.35)	0.810	1.00 (0.77-1.31)	0.99	1.00 (0.77-1.31)	0.99

Abbreviations: CCI, Charlson Comorbidity Index;

* Conditional logistic regression analysis, Significance at P < 0.05

† Stratified model for age, sex, income, and region of residence.

‡ Model 1 was adjusted for SBP, DBP, fasting blood glucose, total cholesterol, hemoglobin, and dyslipidemia.

§ Model 2 was adjusted for model 1 plus obesity, smoking, alcohol consumption, and CCI scores.

S2 table Crude and adjusted odd ratios (95% confidence interval) of date of lipophilic statin prescription (1 year) for hearing impairment with stratified subgroup according age and sex.

Characteristics	Odds ratios for hearing impairment					
	Crude†	P-value	Model 1†‡	P-value	Model 2†§	P-value
Total participants (n = 21,935)						
Lipophilic statin prescription (1 year)	0.98 (0.91-1.07)	0.761	0.95 (0.87-1.04)	0.268	0.95 (0.87-1.04)	0.272
Age < 70 years old (n = 11,910)						
Lipophilic statin prescription (1 year)	1.03 (0.90-1.17)	0.673	0.99 (0.86-1.13)	0.865	0.99 (0.86-1.13)	0.867
Age ≥ 70 years old (n = 10,025)						
Lipophilic statin prescription (1 year)	0.96 (0.86-1.07)	0.458	0.93 (0.83-1.05)	0.218	0.93 (0.83-1.05)	0.223
Men (n = 13,255)						
Lipophilic statin prescription (1 year)	0.95 (0.85-1.07)	0.416	0.91 (0.81-1.03)	0.147	0.91 (0.81-1.03)	0.148
Women (n = 8,680)						
Lipophilic statin prescription (1 year)	1.03 (0.91-1.16)	0.657	0.99 (0.87-1.13)	0.891	0.99 (0.87-1.13)	0.908

Abbreviations: CCI, Charlson Comorbidity Index;

* Conditional logistic regression analysis, Significance at P < 0.05

† Stratified model for age, sex, income, and region of residence.

‡ Model 1 was adjusted for SBP, DBP, fasting blood glucose, total cholesterol, hemoglobin, and dyslipidemia.

§ Model 2 was adjusted for model 1 plus obesity, smoking, alcohol consumption, and CCI scores.

S3 table Crude and adjusted odd ratios (95% confidence interval) of date of hydrophilic statin prescription (1 year) for hearing impairment by severity of hearing impairment

Characteristics	Odds ratios for hearing impairment					
	Crude†	P-value	Model 1†‡	P-value	Model 2†§	P-value
Severe hearing loss (n = 4,075 for hearing impairment, n = 16,300 for control)						
Hydrophilic tatin prescription (1 year)	0.91 (0.76-1.10)	0.341	0.88 (0.73-1.06)	0.167	0.88 (0.73-1.06)	0.170
Profound hearing loss (n = 312 for hearing impairment, n = 1,248 for control)						
Hydrophilic tatin prescription (1 year)	1.13 (0.56-2.27)	0.728	1.17 (0.59-2.35)	0.654	1.27 (0.63-2.57)	0.507

Abbreviations: CCI, Charlson Comorbidity Index;

* Conditional logistic regression analysis, Significance at P < 0.05

† Stratified model for age, sex, income, and region of residence.

‡ Model 1 was adjusted for systolic blood pressure, diastolic blood pressure, fasting blood glucose, total cholesterol, hemoglobin, and dyslipidemia.

§ Model 2 was adjusted for model 1 plus obesity, smoking, alcohol consumption, and CCI scores.

S4 table Crude and adjusted odd ratios (95% confidence interval) of date of lipophilic statin prescription (1 year) for hearing impairment by severity of hearing impairment

Characteristics	Odds ratios for hearing impairment					
	Crude†	P-value	Model 1†‡	P-value	Model 2†§	P-value
Severe hearing loss (n = 4,075 for hearing impairment, n = 16,300 for control)						
Lipophilic tatin prescription (1 year)	0.99 (0.91-1.08)	0.815	0.95 (0.86-1.04)	0.262	0.95 (0.87-1.04)	0.263
Profound hearing loss (n = 312 for hearing impairment, n = 1,248 for control)						
Lipophilic tatin prescription (1 year)	0.92 (0.59-1.42)	0.703	0.98 (0.62-1.54)	0.920	0.95 (0.60-1.50)	0.827

Abbreviations: CCI, Charlson Comorbidity Index;

* Conditional logistic regression analysis, Significance at P < 0.05

† Stratified model for age, sex, income, and region of residence.

‡ Model 1 was adjusted for systolic blood pressure, diastolic blood pressure, fasting blood glucose, total cholesterol, hemoglobin, and dyslipidemia.

§ Model 2 was adjusted for model 1 plus obesity, smoking, alcohol consumption, and CCI scores.
